# GM-CSF/IL-3/IL-5 receptor common β chain (CD131) expression as a biomarker of antigen-stimulated CD8^+ ^T cells

**DOI:** 10.1186/1479-5876-6-17

**Published:** 2008-04-15

**Authors:** Silvia Selleri, Sara Deola, Zoltan Pos, Ping Jin, Andrea Worschech, Stefanie L Slezak, Cristiano Rumio, Monica C Panelli, Dragan Maric, David F Stroncek, Ena Wang, Francesco M Marincola

**Affiliations:** 1 Infectious Disease and Immunogenetics Section (IDIS), Department of Transfusion Medicine, Clinical Center, National Institutes of Health, Bethesda, MD, USA; 2 Hematology and BMT Unit, Scientific Institute H S. Raffaele, Milan, Italy; 3 Cell Processing Section, Department of Transfusion Medicine, Clinical Center, National Institutes of Health, Bethesda, MD, USA; 4 Department of Human Morphology, Universitá degli Studi di Milano, Milan, Italy; 5 Department of Medicine, University of Pittsburgh Cancer Center, Pittsburg, PA, USA; 6 Laboratory of Kidney and Electrolyte Metabolism, NHLBI, NIH, Bethesda, MD, USA

## Abstract

**Background:**

Upon Ag-activation cytotoxic T cells (CTLs) produce IFN-γ GM-CSF and TNF-α, which deliver simultaneously pro-apoptotic and pro-inflammatory signals to the surrounding microenvironment. Whether this secretion affects in an autocrine loop the CTLs themselves is unknown.

**Methods:**

Here, we compared the transcriptional profile of Ag-activated, Flu-specific CTL stimulated with the FLU M1:58-66 peptide to that of convivial CTLs expanded *in vitro *in the same culture. PBMCs from 6 HLA-A*0201 expressing donors were expanded for 7 days in culture following Flu M1:58-66 stimulation in the presence of 300 IU/ml of interleukin-2 and than sorted by high speed sorting to high purity CD8+ expressing T cells gated according to FluM1:58-66 tetrameric human leukocyte antigen complexes expression.

**Results:**

Ag-activated CTLs displayed higher levels of IFN-γ, GM-CSF (CSF2) and GM-CSF/IL-3/IL-5 receptor common β- chain (CD131) but lacked completely expression of IFN-γ receptor-II and IFN-stimulated genes (ISGs). This observation suggested that Ag-activated CTLs in preparation for the release of IFN-γ and GM-CSF shield themselves from the potentially apoptotic effects of the former entrusting their survival to GM-SCF. *In vitro *phenotyping confirmed the selective surface expression of CD131 by Ag-activated CTLs and their increased proliferation upon exogenous administration of GM-CSF.

**Conclusion:**

The selective responsiveness of Ag-activated CTLs to GM-CSF may provide an alternative explanation to the usefulness of this chemokine as an adjuvant for T cell aimed vaccines. Moreover, the selective expression of CD131 by Ag-activated CTLs proposes CD131 as a novel biomarker of Ag-dependent CTL activation.

## Background

*In vivo *animal models suggest that the activation of CD8-expressing cytotoxic T cells (CTLs) follows a linear pattern in which an expansion phase occurring within the first week after Ag stimulation rapidly evolves into a contraction phase in which surviving memory CTLs resume a quiescent phenotype [1,2]. During the expansion phase, Ag-activated CTLs boast a robust enhancement of effector functions including the activation of cytotoxic mechanisms and the production of pro-inflammatory cytokines such as interferon (IFN)-γ. It is believed that such activation occurs through signaling associated with the Ag-specific triggering of the T cell receptor (TCR) combined with other co-stimulatory signals. In summary, naïve and, to a certain degree, long-term memory CTL activation and expansion is dependent upon three types of stimulation [3]; the first is the direct interaction between the TCR and the major histocompatiblity (MHC)/epitope complex. This interaction determines the specificity of the activation. However, TCR triggering is not sufficient by itself to sustain a forceful activation and expansion of CTLs and it may lead to unresponsiveness if others stimulatory signals are not provided simultaneously. A second signaling requirement is absolved by cell-to-cell interactions involving co-stimulatory molecules expressed on the surface of Ag-presenting cells. This interaction may sustain a few cell divisions but is insufficient to induce clonal expansion and full activation of effector functions. Thus, a third signal is needed, which is provided by immune-modulatory cytokines released by Ag-presenting cells, helper T cells or other immune cells in response to pro-inflammatory signals provided by pathogens or other environmental conditions. This third signal can be modeled experimentally by the exogenous administration of pro-inflammatory cytokines such as interleukin (IL)-2 [4].

Recombinant human IL-2 has been extensively used for the selective *in vitro *expansion of CTLs naturally exposed *in vivo *to Ag such as tumor infiltrating lymphocytes [5] or vaccine-induced circulating lymphocytes [6]. The *in vitro *expansion of CTLs exposed to Ag *in vivo*, strictly requires cytokine stimulation (as exemplified by IL-2); furthermore, *in vitro *stimulation in the presence of IL-2 leads not only to selective expansion of Ag-specific CTLs but also to the activation of their effector functions [4] paralleling the expansion phase described in other experimental models [1,7].

Segregating the respective contribution of Ag-specific signaling and environmental co-stimulation within the same microenvironment may provide useful insights about the mechanisms involved in the selective activation of Ag-exposed CTLs within a T cell population and shed light on the requirements for full activation of CTL effector functions in the target organ during distinct immune reactions including tumor regression following immunotherapy [8,9], acute allograft rejection [10], clearance of viral infection [11] and flares of autoimmunity [12].

In a simplified *in vitro *model of human CTL activation, we previously observed that neither Ag-stimulation in the presence of signal two nor the presence of signal 3 alone could induce *in vitro *expansion and activation of Ag-exposed CTLs and only the combination of the three could induce effective CTL responses [4]. Analysis of the transcriptional patterns associated with the complete activation of effector CTL responses suggested that proliferation and effector function were both dependent upon the combined presence of the three signals. However, further dissection of transcriptional patterns induced by the administration of IL-2 to peripheral blood mononuclear cells (PBMC) or non Ag-activated CD4 and CD8 T cell sub-populations suggested that the effects of IL-2 on T cell signaling are powerful but non-specific in the absence of TCR triggering [13]. Thus, to discriminate the individual contribution of direct TCR triggering on CTL activation, we compared the transcriptional profile of Ag-exposed CTLs to non-Ag-exposed, non-proliferating CTLs sharing identical environmental conditions. The model evaluated the kinetics of proliferation of HLA-A*0201-restricted, Flu Matrix protein epitope M1:58-66-specific CTLs; seven days following *in vitro *Ag stimulation with M1:58-66 and *in vitro *culture in 300 IU of human recombinant IL-2 (Novartis-Chiron CO, Emeryville CA), we separated with tetrameric flu-specific human leukocyte antigen/complexes (tHLA) proliferating CTLs from their companions CD8 expressing T cells (convivial CTLs).

## Materials and methods

### Transcriptional characteristics of stimulated versus resting CD8 expressing T cells

#### In vitro sensitization (IVS)

PBMCs were obtained by leukapheresis from HLA-A*0201-expressing normal volunteers and frozen after Ficoll separation. HLA-A*0201 expression was documented by sequence-based typing [14]. PBMCs from 6 donors were thawed and plated in complete Iscove medium (Life Technologies, Grand Island, NY) supplemented with 10% heat inactivated human AB serum, 10 mM HEPES buffer, 100 U/ml penicillin-streptomycin, 0.5 mg/ml amphotericin B and 0.03% glutamine, at the density of 10^6 ^cells/well in 48 multiwell plate. After overnight panning, cells were pulsed at day 1 with 1 μM Flu M1:58-66 peptide (Princeton Biomolecules, Langhorne, PA) and the following day human recombinant IL-2 300 IU/ml (rHuIL-2, Chiron Co, Emeryville, CA) was added. IL-2 was added every two days. At day 1, T cells were stained with Carboxy Fluoroscein Succinimidyl Ester (CFSE) to monitor their proliferation. PBMC cultures were continued for 7 days till sorting.

### Cell sorting

**Figure 1:**
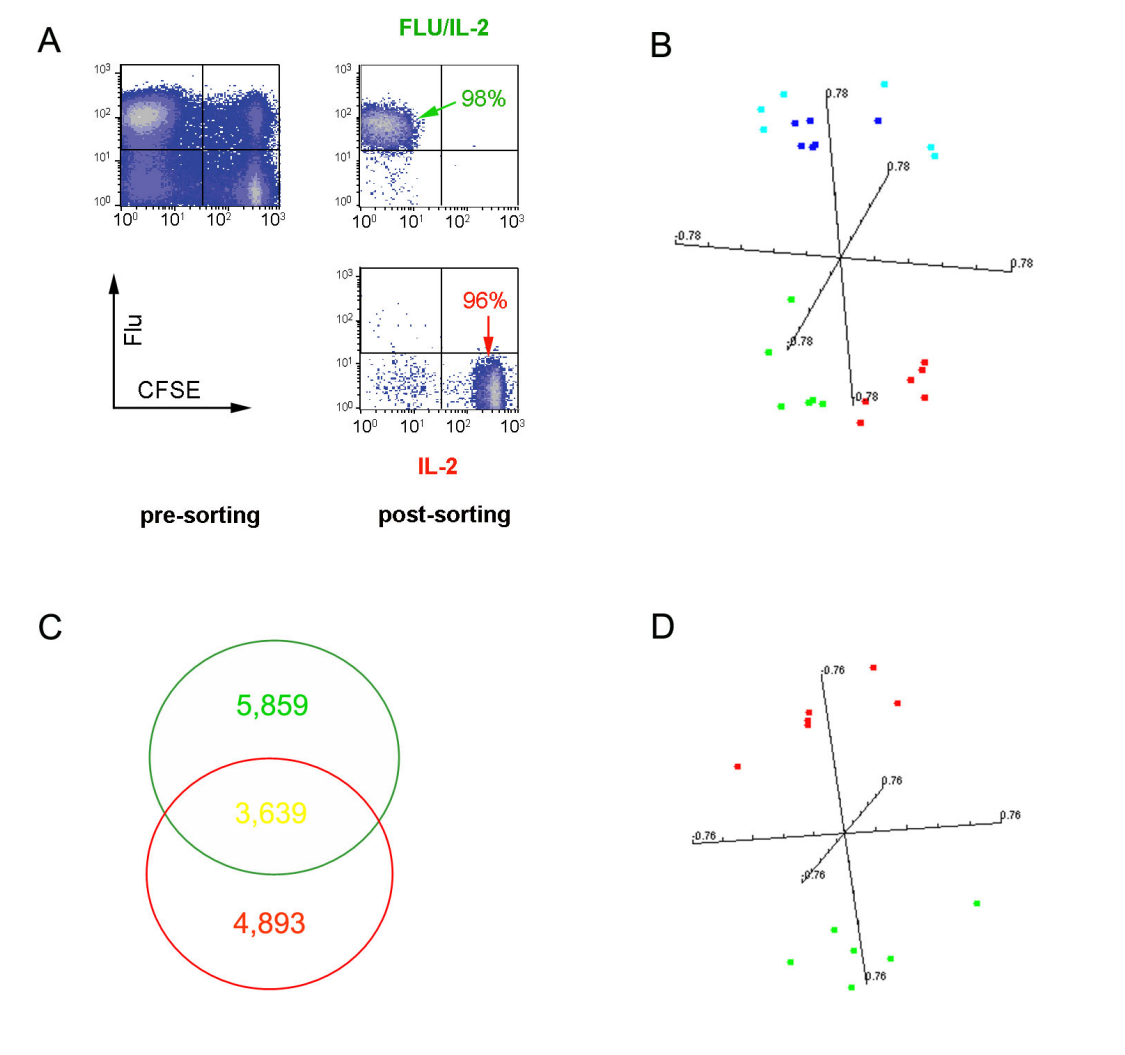
**Gene expression profile: comparison between antigen specific and convivial non specific CD8+ T cells**. **A) **representative example of *in vitro *expansion of Flu M1:58-66-specific CD8 T cells in the presence of 300 IU/IL-2 and purity of the sorting procedure by high speed flow cytometry (FACSVantage SE); **B) **multiple dimensional scaling representing the four populations of CD8 T cells based on the complete data set of 16,726 cDNA clones. CD8 expressing, CSFE^high^, Flu/tHLA negative T cells (non Flu specific) are referred to as IL-2 while CD8 expressing, CSFE^low^, Flu/tHLA positive T cells are referred to as IL-2+Flu; **C) **Venn diagram displaying the extent of overlap between genes differentially expressed by Flu-specific and non Flu-specific CTLs from the same culture compared to quiescent CD8-expressing T cells; **D) **Multiple dimensional scaling plot representing Flu-specific and convivial CD8 T cells distribution using the complete data set of 16,726 cDNA clones.

On the eight day in culture, CD8-expressing T cells were enriched by negative selections using magnetic beads before sorting (Miltenyi Biotec, Auburn, CA) on an autoMACS separator. Median purity of CD8 T cells eluted from the columns was 85%. Sorting was then performed by high speed flow-cytometry (FACSVantage SE, BD); a logical gate was applied on SSC and live/dead staining with DAPI to check the viability of sorted cells. The sorting was always done in the Normal-R mode, which optimizes for cell purity, as confirmed by re-analysis of the sorted populations. Sorting was based according to level of tHLA-Flu and CFSE staining of CD8-expressing T cells segregating the tHLA-Flu+/CFSE- proliferating cells from the tHLA-Flu-/CFSE+ non proliferating CTLs. Median purity of Flu-specific and non-Flu-specific CTLs was above 95 % in all experiments (Figure 1A). For the various sorting procedures the following monoclonal antibodies were used: CD8-PE or CD8-FITC, CD3-PerCP (all from BD Biosciences Pharmingen, San Diego, CA). As negative control cells were stained with IgG FITC or PE conjugated, according to the respective antibody's isotype. Cells were analyzed by FACS sort (BD Bioscience) gating them on living CD3-expressing lymphocytes.

### RNA handling for transcriptional profiling

Total RNA was isolated with RNeasy minikits (Qiagen, Valencia, CA) and amplified into anti-sense RNA as previously described [15,16]. First strand cDNA synthesis was accomplished in 1μl SUPERase·In (Ambion, Foster City, CA) and ThermoScript RT (Gibco-Invitrogen, Carlsbad, CA) in 2 μg bovine serum albumin. RNA quality was verified by Agilent technologies (Palo Alto). Anti-sense RNA was labeled with Cy5-dUTP (Amersham, Piscataway, NJ) and co-hybridized with reference pooled normal donor peripheral blood mononuclear cells (PBMC) labeled with Cy3-dUTP to custom made 17K-cDNA array platform (UniGene cluster) printed at the Infectious Disease and Immunogenetics Section, DTM, CC, NIH with a configuration of 32 × 24 × 23 and contained 17,500 elements. Clones used for printing included a combination of the Research Genetics RG_HsKG_031901 8 k clone set and 9,000 clones selected from the RG_Hs_seq_ver_070700 40 k clone set. The 17,500 spots included 12,072 uniquely named genes, 875 duplicated genes and about 4,000 expression sequence tags.

Arrays were scanned on a GenePix 4000 (Axon Instruments) and analyzed using BRB-ArrayTools Version: 3.3, Cluster and Tree View software.

### Phenotyping and proliferation of Ag-activated CTLs

#### Cell culture

PBMCs from HLA-A*0201 healthy volunteers were obtained from leukapheresis by Ficoll separation and kept frozen in liquid nitrogen in aliquots of 10^8 ^cells/vial. At day 0, PBMCs (1 vial/donor) were thawed and plated in 24 mw plates (2 × 10^6 ^cells/well). After resting overnight, 10^5 ^cells/donor were stained in order to determine the percentage of CD8+ FLU+ T cells at day 1. All the remaining cells were stimulated with Flu peptide (Flu M1:58-66, 1 μg/ml, Princeton Biomolecules, Langhorne, PA). The day after cells in culture were harvested, counted and plated in new 24 mw plates (2 × 10^6 ^cells/well) and treated as follows: 1) untreated (Flu stimulation on day 1 only); 2) IL-2 (300 U/ml, Chiron, Emeryville CA); 3) IL-2+GM-CSF (10^3 ^U/ml, PreProtech, Rocky Hill, NJ); 4) GM-CSF; 5) IL-2+IFN-γ (500 U/ml, Actimmune, Brisbane, CA); 6) IFN-γ. Cytokines were added every 2 days, replacing each time half of the medium to avoid their accumulation in the supernatant. At day 6 and 12, cells from 1 well/treatment were harvested, counted and stained for FACS analysis.

#### FACS analysis

Harvested cells were washed with buffer and stained with t-FLU-PE (FLU M1 iTAg MHC Tetramer, Beckman Coulter, Miami, FL), mouse IgG1k anti CD8 PE-Cy5 (Becton Dickinson, Franklin Lakes, NJ), mouse IgG2a anti GM-CSF-R (by Millipore, Billerica, MA, detected by using a secondary antibody against mouse IgG2a Alexa 647 conjugated, from Invitrogen, Carlsbad, CA), mouse IgG1k anti IFN-γ Receptor β chain (Abcam, detected by using a secondary antibody against mouse IgG1k Alexa 488 conjugated, from Invitrogen). In order to avoid the reaction of the secondary antibody used for IFNγ detection with the Fc of the IgG1k anti CD8, the staining for IFNγ receptor was performed separately and anti CD8 was added after washing as last step.

FACS analysis was performed using a FACScalibur by BD Pharmingen.

### Statistical analysis

#### Transcriptional profiling

The raw data were filtered to exclude spots with minimum intensity by arbitrarily setting a minimum intensity requirement of 300 in both fluorescence channels. If the fluorescence intensity of one channel was over and that of the other below 300 the fluorescence of the low intensity channel was arbitrarily set to 300. Spots with diameters < 25 μm and flagged spots were excluded from the analysis. The filtered data were then normalized using the lowess smother correction method. All statistical analyses were performed using the log_2_-based ratios normalizing the normal value in the array equal to zero.

Validation and reproducibility were measured using an internal reference concordance system based on the expectation that results obtained through the hybridization of the same test and reference material in different experiments should perfectly collimate. The level of concordance was measured by periodically re-hybridizing the melanoma cell line A375-melanoma (American Type Culture Collection, Rockville MD) to the reference samples consisting of pooled PBMCs as previously described [17]. This analysis demonstrated a higher than 95% concordance level. Non-concordant genes were excluded from subsequent analysis.

Supervised class comparison utilized the BRB ArrayTool [18] developed at NCI, Biometric Research Branch, Division of Cancer Treatment and Diagnosis. Paired samples were compared with a two-tailed paired Student *t *test. Unpaired samples were tested with a two-tailed un-paired Student *t *test assuming unequal variance or with an F test as appropriate. All analyses were tested for a univariate significance threshold set at a p_2_-value < 0.005. Gene clusters identified by the univariate *t *test were challenged with two alternative additional tests, a univariate permutation test (PT) and a global multivariate PT. The multivariate PT was calibrated to restrict the false discovery rate to 10%. Genes identified by univariate *t *test as differentially expressed (p_2_-value < 0.005) and a PT significance < 0.05 were considered truly differentially expressed. Gene function was assigned based on Database for Annotation, Visualization and Integrated Discovery (DAVID) and Genontology. Multiple dimensional scaling was performed using the BRB Array tool.

#### Functional studies

Fold increase in CD8+ FLU+ T cells was based on the calculation of the absolute number of CD8 expressing T cells at day 1, day 6 and day 12. Their fold increase (FI) was calculated by dividing their number at day 6 and 12 over their starting number at day 1. The same assessment was done for flu-positive and flu-negative CTLs. Average and standard error from the mean (SEM) are presented as appropriate. A paired Student *t *test was applied to calculate level of significance.

## Results and discussion

### Global Differences between Ag-stimulated, IL-2-activated and quiescent CD8 expressing T cells

There were extensive differences between quiescent CD8 expressing T cells analyzed *ex vivo *and their counterparts maintained in *in vitro *culture in the presence of 300 IU IL-2 and Ag stimulation. An univariate F-test with random variance model identified 4,702 clones out of 16,726 present in the array to be differentially expressed at a p-value < 0.001. The probability of randomly obtaining this number of genes at the selected level of significance (p < 0.005) if there were no real differences among groups was calculated as 0 by multivariate permutation test (Table 1). To assess for possible effects due to the *in vitro *culture conditions, we maintained PBMCs in culture for 24 hours in the absence of IL-2 and sorted CD8 expressing T cells before transcriptional profiling. This control demonstrated high similarity of *in vitro *maintained CD8 T cells with the profile of *ex vivo *analyzed CD8 T cells. Thus, the functional genomics changes observed in *in vitro *stimulated CD8 T cells are specific to stimulation and not simply due to *in vitro *culture artifacts. Multiple dimensional scaling based on the global 16,726 cDNA clone data set clearly separated the *ex vivo *and *in vitro *quiescent from the *in vitro *activated populations (Figure 1B).

**Table 1:** Difference in the transcriptional pattern of quiescent compared to stimulated CD8 T cells

**Class Comparison**	**# of genes differentially expressed at Student *t *test or *F *test p_2_-value < 0.001**	Permutation Test (p-value) **Multivariate**
***Ex vivo*vs CD8 in culture vs IL-2 vs IL-2+Flu**	6,692 (*F *test, n = 24)	0
***Ex vivo*vs combined IL-2 and IL-2+Flu**	5,527 (*t *test, n = 18)	0
***Ex vivo*vs IL-2 vs IL-2+Flu**	4, 702 (*F *test, n = 18)	0
***Ex vivo*vs IL-2**	4,893 (*t *test, n = 12)	0
***Ex vivo*vs IL-2+Flu**	5, 859 (*t *test, n = 12)	0
**IL-2 vs IL-2+Flu**	1,727 (t test), n = 12	0

*Ex vivo *refers to the transcriptional analysis of freshly isolated and processed CD8 positive T cells; CD8 T cells were also sorted by high speed FACS sorter from PBMC from HLA-A*0201 expressing normal donors that had been cultured for 7 days in the presence of 300 IU IL-2 following stimulation with the Flu M1:58-66 peptide according to their level of CFSE and Flu/tHLA staining. CD8 expressing, CSFE^high^, Flu/tHLA negative T cells (non Flu specific) are referred to as IL-2 while CD8 expressing, CSFE^low^, Flu/tHLA positive T cells are referred to as IL-2+Flu

Analysis of individual experimental conditions against each other demonstrated that the biggest differences in transcriptional patterns were present between the Flu-specific CTLs stimulated *in vitro *and the quiescent *ex vivo *CD8 T cells (three way *t *test) with the least differences noted between the Flu-specific CTLs and the convivial non-Flu-specific CTLs from the same culture.

### Transcriptional patterns shared by Ag-specific and convivial CTLs compared to quiescent *ex vivo *analyzed CD8-expressing T cells

The transcriptional pattern of *in vitro *stimulated CTLs whether exposed to Ag (Flu-specific CD8 T cells) or only to IL-2 was similar relative to that of *ex vivo *isolated or *in vitro *maintained CD8 T cells. In particular, multiple dimensional scaling based on the complete 16,726 gene data set clearly separated the unstimulated from the stimulated populations. Of 5,859 genes differentially expressed between Flu-specific CTLs and *ex vivo *CD8 T cells, 3,639 were concordantly expressed by convivial CTLs (Figure 1C). Thus, CTLs maintained in the same culture demonstrate similar transcriptional patterns independent of their exposure to Ag-specific stimulation. Among the genes similarly up-regulated in both subgroups of stimulated T cells were perforin, granzyme A, TNF-α and the IL-2 receptor α chain. Overall, the transcriptional profile of the genes concordantly expressed by *in vitro *stimulated CTLs was similar to that of our previous reported analysis [13].

### Transcriptional patterns specific to Ag-specific activation

The aim of this study was to identify those signatures that are determined by the long term effects of Ag stimulation independent of other co-existing factors that may influence the activation and function of CD8+ T cells. For this reason, we focused our analysis on genes that were differentially expressed between CFSE^high^, Flu/tHLA positive CD8 T cells and CSFE^low^, Flu/tHLA negative CD8 T cells. An unpaired *t *test identified 1,727 genes to be differentially expressed between the two populations at a p_2_-vlue < 0.001 (Table 1). It should be clarified that several of these genes were concordantly differentially expressed in both subpopulations compared with quiescent *ex vivo *isolated CD8-expressing T cells. However, the degree in which the expression was altered in the two subsets was sufficiently different to result in significant differences between the two populations. The differences identified were significant according to the multivariate permutation test (p-value = 0). Multiple dimensional scaling analysis based on the complete date set confirmed the separation of the two populations (Figure 1D).

Among the genes differentially expressed by the two populations 644 were up-regulated in Ag-specific CTLs compared to convivial CTLs. The rest (1,083) were down-regulated. The annotations related to biological functions derived through gene ontology suggested that the genes that were predominantly up-regulated in Ag-specific CTLs belong to several categories. Although some categories appeared particularly enriched, they contained a relatively small number of genes, while the categories with the largest absolute number of genes included: cell cycle and cell division (111 genes), response to endogenous stimulus (45 genes) and cytokine production (17 genes). Gene Ontology analysis suggested, therefore, that even seven days after the original stimulus the predominant differences between Ag-exposed Flu-specific CTLs and their culture companions were related to a broader activation of pro-proliferative stimuli, signaling and cytokine production in the former.

Gene Ontology was also applied to identify genes associated with immunological functions; this analysis identified 58 (expected number 52.8; observed over expected ratio = 1.10) up-regulated in the Flu-specific CTLs and 212 out of 988 (expected number 80.3; observed over expected ratio = 2.64) down-regulated relative to the convivial CTLs (Fisher test p_2_-value < 0.001).

**Figure 2:**
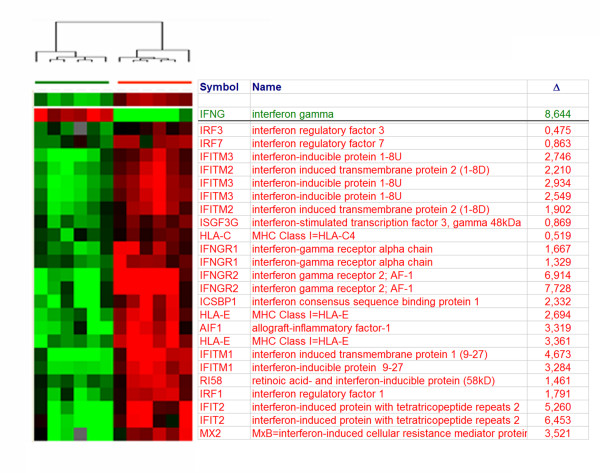
**Gene expression profile: comparison between antigen specific and convivial non specific CD8+ T cells**. **A) **Selection of genes among 1,727 genes differentially expressed between Flu-specific (green bar) and convivial (red bar) CD8 T cells at a *t *test p_2_-value < 0.001 whose annotation includes the word "interferon". The table displays the gene symbol, its name and the level of differential expression (Δ) as the CY5/CY3 ratio of fly-specific versus convivial in green and vice versa in red.

The immunologically-related genes most expressed by Ag-activated or convivial CTLs are shown in Table 2. The transcript most abundant in Ag-specific CTLs was IFN-γ (Figure 2). Conversely, IFN-γ receptor 2 (β-chain) ranked highest among the immune genes up-regulated bye convivial CTLs, which paralleled the over-expression of interferon-stimulated genes (ISGs) selectively in these cells while ISGs where completely shut off in Ag-activated CTLs. The lack of expression of ISGs by Ag-activated CTLs was associated with lack of expression of the IFN-γ receptor α and β and the IFN-γ receptor accessory factor AF-1. This observation suggested that Ag-activated, terminally differentiated CTLs shelter themselves from the potentially harmful autocrine effects of IFN-γ. This observations supports the lack of responsiveness of Ag-specific T cells to IFN-γ during the expansion phase which is slowly regained during the contraction phase previously reported in an experimental animal model [19]. On the other hand, Ag-stimulated CTLs expressed higher levels of CSF2 receptor β chain, (GM-CSF/IIL-5/IL-3 receptor common β-chain, CSFR2B, CD131), chemokine (C-X-C motif) receptor 6 (CXCR6), C-C chemokine receptor 1 (CCR1) and IL-2R α- and β-chains. In addition, Ag-stimulated CTLs strongly down-regulated the chemokine (C-C motif) receptor 7 in accordance with their effector T cell differentiation. Interestingly, the over-expression of CD131 was associated with very high expression of its ligand colony stimulating factor 2 (CSF2, GM-CSF) suggesting that this autocrine loop may play an important role in promoting their survival. Moreover, several cytokines were produced including chemokine (C-C motif) ligand 3 (CCL3, MIP-1α), ligand 4 (CCL4, MIP-1β) and ligand 18 (CCL18, PARC).

**Table 2:** 

	Immune Genes Up-regulated in Flu-specific CD8 T cells			Immune GenesUp-regulated in convivial CD8 T cells	
Symbol		Δ	Symbol		Δ
IFNG	Interferon gamma	8.64	IFNGR2	interferon-γ receptor 2	7.73
CSF2	Colony stimulating factor 2	7.55	GZMK	granzyme K	7.12
CCL3	Chemokine (C-C motif) ligand 3	7.13	XCL1	Chemokine (C motif) ligand 1	6.39
CD38	CD38	6.99	SOCS3	STAT induced STAT inhibitor-3 = CIS3	5.02
CD80	CD80 = B7-1	6.90	CX3CR1	Chemokine (C-X3-C motif) receptor 1	4.91
CSF2RB	Colony stimulating factor 2 receptor, beta	5.58	CCR7	Chemokine (C-C motif) receptor 7	4.58
APOA2	Apolipoprotein A-II	5.13	TIMP2	TIMP metallopeptidase inhibitor 2	4.53
GZMB	Granzyme B	4.72	IL24	interleukin 24	4.47
CSF2RB	GM-CSF/IL-5/IL-3 receptor common beta chain	3.64	PF4	Platelet factor 4 (chemokine) ligand 4	4.29
GNLY	Granulysin	3.62	LY86	Lymphocyte antigen 86	4.26
CCL4	MIP-1 beta	3.54	ITGA6	CD49F = Integrin alpha 6	3.75
CD86	CD86 = B7.2	3.54	PECAM1	platelet/endothelial cell adhesion molecule (CD31)	3.37
CD226	adhesion molecule DNAM-1	3.51	LY9	lymphocyte antigen 9	2.97
CXCR6	Chemokine (C-X-C motif) receptor 6	3.32	JAK1	Janus kinase 1	2.68
ITGA2	Integrin, alpha 2 (CD49B)	3.30	TNFSF13	Tumor necrosis factor superfamily13	2.68
DUSP16	Dual specificity phosphatase 16	2.91	IL8	Interleukin 8	2.55
IL1RAP	Interleukin 1 receptor accessory protein	2.37	TIMP1	tissue inhibitor of metalloproteinase 1	2.47
CCR1	C-C chemokine receptor 1	2.30	CISH	Cytokine inducible SH2-containing protein	2.46
DUSP5	dual specificity phosphatase 5	2.27	CD163	CD163	2.39
ITGB7	CD103 beta = Integrin beta 7	2.22	CTSS	Cathepsin S	2.17
ICOS	inducible T-cell co-stimulator	2.22	GABBR1	GABA-BR1a (hGB1a) receptor	2.16
DUSP10	Dual specificity phosphatase 10	1.88	SELL	CD62L = L-selectin	2.16
GZMA	Granzyme A	1.43	CXCL1	GRO1 = GRO α	2.15
CTSD	Cathepsin D	1.43	CSF3R	G-CSF receptor	2.14
ENTPD1	CD39	1.31	GRN	Granulin	2.06
CCL18	PARC	1.10	CD33	CD33 molecule	2.04
CD58	LFA-3	1.10	IL11RA	IL-11 receptor α chain	1.90
IL2RB	IL-2 receptor beta chain	1.07	CXCR4	chemokine (C-X-C motif), receptor 4 (fusin)	1.85
CCL2	MCP-1	1.06	IL7R	IL-7 receptor α chain	1.58
CTSC	Cathepsin C	0.98	VEGFB	Vascular endothelial growth factor B	1.55
IL2RA	interleukin 2 receptor, alpha	0.92	IL4R	IL-4 receptor α chain	1.45
			STAT1	STAT1	1.39
			IFNGR1	interferon-gamma receptor α chain	1.33

Ag-activated CTLs also expressed higher levels of granzyme B and to a lesser degree granzyme A while their convivial counterparts expressed high levels of granzyme K. As previously observed, Perforin, was strongly and equally un-regulated in both populations compared to quiescent CD-expressing T cells studied *ex vivo *or *in vitro *[4].

Finally, Ag-activated CTLs expressed higher levels of the co-stimulatory molecules CD80 and CD86.

### GMCSF effects on Ag-specific CTLs

The high levels of IFN-γ and GMSCF transcript (but not protein) expression together it the up-regulation of CMCSF receptor β chain (CD131) and the down regulation of the IFN-γ receptors I and II by Ag-activated CTLs suggested the intriguing possibility of a bipolar relationship of Ag-activated CTLs with the potential autocrine effects of the two cytokines. It appears that Ag-activated CTLs shield themselves from the potential harmful affects of IFN-γ [20] while accepting the potentially proliferative support of GM-CSF; a growth factor without known pro-apoptotic functions [21]. This bipolar behavior may explain the selective survival and expansion of Ag-stimulated CTLs *in vitro *(an potentially *in vivo*).

**Figure 3:**
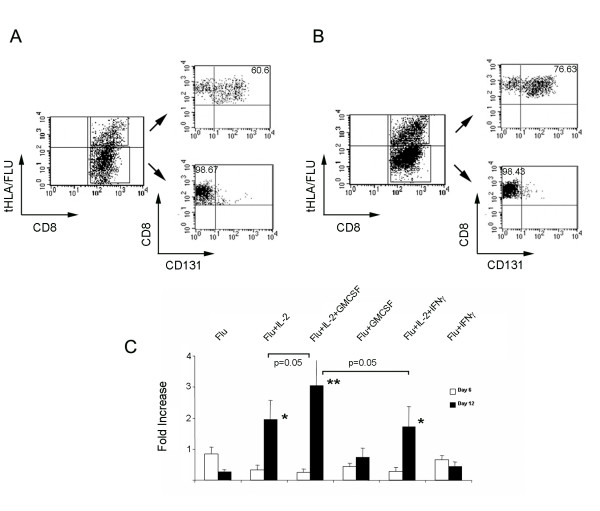
**CD131 expression following IVS with Flu M1:58-66 peptide**. Expression of CD131 (GMCSF receptor β chain) in Flu M1:58-66-activated (tHLA/Flu +) and convivial (tHLA/Flu-) CTLs (corresponding to R4 and R7 in **Figure 1**) after 6 (**A**) and 12 (**B**) days of IVS of HLA-A*0201 expressing PBMCs in the presence of 300 IU/ml of IL-2; **C **– Fold increase (FI) of HLA-A*0201 PBMCs exposed to IVS with Flu M1:58-66 peptide (Flu) plus exogenous administration every other day of IL-2 (300 IU/ml), GMCSF (1,000 U/ml), IFN-γ (500 IU/ml) or a combination of them for 6 (white bars) or 12 (black bars) days. Data are presented as average ± standard error from the mean of 5 experiments performed in PBMCs from 5 different donors; * and ** = paired Student *t *test p-value ≤ 0.05 and ≤ 0.01 respectively between comparing FI in individuals experimental groups with the FI in respective PBMC populations stimulated only with Flu; The difference in significance between Flu+IL-2+GMCSF FI at day 12 and that of Flu+IL-2 and Flu+IL-2+IFN-γ are outlined specifically in the graph.

To test the potential of such hypothesis, we analyzed CD131 expression by Flu-specific CTLs. As shown in Figure 3, at day 6 and 12 Flu-specific CTLs consistently express CD131 which is totally absent in the convivial CTLs. IVS of HLA-A*0201-expressing PBMCs with 1 μM Flu M1:58-66 peptide in the presence of IL-2, GMCSF, IFN-γ or a combination of the them with IL-2 demonstrated that while GMCSF (1,000 U/ml) selectively enhances the expansion of Flu-specific CTLs after 12 days in culture when combined to IL-2 (300 IU/ML) administration while IFN-γ (500 U/ml) has no effect consistent with their lack of expression of the IFN receptors (Figure 3C). Not the combination IL-2+GMCSF nor other cytokine combination exerted any effect on the proliferation of convivial CTLs (data not shown).

In conclusion, this preliminary study suggests that, at least during IVS, preferential survival/expansion of Ag-activated CTL may be partially mediated through a bipolar regulation of their sensitivity to the autocrine secretion of cytokines; IFN-γ and GMCSF appear to play a dominant role at this junction as other two cytokines known to be produced by activated CTLs (TNF-α and IL-2) where similarly highly expressed at the transcriptional level by both Ag-activated and convivial CTLs. The confirmation of the selective expression of CD131 on the surface of Ag-activated CTLs and its likely functional association with the selective response of Ag-activated CTLs to exogenous GM-CSF suggests a previously unreported positive feed back autocrine loop that may stimulate CTL growth in response to further Ag stimulation [21]. In addition, the positive role that GMCSF may play in the proliferation of CD131-expressing Ag-activated CTLs, may explain the beneficial effects of this cytokine used as vaccine adjuvant, which has been so far attributed exclusively to its role in activating and maturing antigen presenting cells [22]. Finally, the selective expression of CD131 by Ag-activated CTLs may qualify this surface marker as a non Ag-specific biomarker of Ag-specific CTL activation. Although extensive *in vivo *and *in vitro *validation is required to support such hypotheses, we believe that the novelty and the potential biological implications of these findings warrant a preliminary disclosure.
